# *Gibberellic acid sensitive dwarf* encodes an ARPC2 subunit that mediates gibberellic acid biosynthesis, effects to grain yield in rice

**DOI:** 10.3389/fpls.2022.1027688

**Published:** 2022-12-22

**Authors:** Tae Young Um, So Yeon Hong, Ji Sung Han, Ki Hong Jung, Sunok Moon, Beom-Soon Choi, Prakash Basnet, Young Soo Chung, Seon Woo Lee, Won Tae Yang, Doh Hoon Kim

**Affiliations:** ^1^ Department of Agriculture and Life Industry, Kangwon National University, Chuncheon, Republic of Korea; ^2^ College of Life Science and Natural Resources, Dong-A University, Busan, Republic of Korea; ^3^ Graduate School of Green-Bio Science, Kyung Hee University, Yongin, Republic of Korea; ^4^ Research Institute, NBIT Co., Ltd., Chuncheon, Republic of Korea

**Keywords:** gibberellic acid, actin-related protein, *Oryza sativa*, rice growth, grain yield

## Abstract

The plant hormone gibberellic acid (GA) is important for plant growth and productivity. Actin-related proteins (ARPs) also play central roles in plant growth, including cell elongation and development. However, the relationships between ARPs and GA signaling and biosynthesis are not fully understood. Here, we isolated *OsGASD*, encoding an ARP subunit from rice (*Oryza sativa*), using the Ac/Ds knockout system. The *osgasd* knockout (Ko) mutation reduced GA3 content in shoots as well as plant growth and height. However, GA application restored the plant height of the *osgasd* Ko mutant to a height similar to that of the wild type (WT). Rice plants overexpressing *OsGASD* (Ox) showed increased plant height and grain yield compared to the WT. Transcriptome analysis of flag leaves of *OsGASD* Ox and *osgasd* Ko plants revealed that OsGASD regulates cell development and the expression of elongation-related genes. These observations suggest that *OsGASD* is involved in maintaining GA homeostasis to regulate plant development, thereby affecting rice growth and productivity.

## Introduction

Plant architecture is important for the productivity of rice (*Oryza sativa*), a major crop worldwide. Rice productivity is mainly determined by a few major factors such as plant height, number of panicles, filling rate, number of spikelets per panicle, total grain weight, thousand-grain weight, and grain size (Walter et al., 2009; Li and Li, 2016). The most important of these is total grain weight, which is determined by grain size and filling rate. Grain size and filling rate in rice are controlled by several regulatory factors (Li et al., 2018; Shi et al., 2020; Jiang et al., 2022). The development of tissues and organs such as flowers and leaves is closely related. Flag leaves provide nutrients *via* photosynthesis, which are crucial to floral organ and seed development (Saitoh et al., 2002; Fujita et al., 2013; Makino et al., 2022). For example, transgenic plants overexpressing *SPIKELET NUMBER* (*SPIKE*) showed increased flag leaf size and spikelet number, with a 13–36% higher yield than wild-type (WT) plants. Plant morphology is determined by cell development, which strongly affects photosynthesis and metabolic activity (Fujita et al., 2013).

Actin is a conserved protein with important roles in various physiological processes (Diao and Huang, 2021). Actin-related proteins (ARPs) are chromatin-modifying complexes involved in diverse intracellular processes. The ARP2/3 complexes comprise various subunits (ARPC1 to ARPC5) that contribute to nucleation during plant cell wall synthesis (Pratap Sahi et al., 2018; García-González et al., 2020). ARPC2 is an important ARP2/3 subunit that interacts with filaments and microtubules in the cytoskeleton, thereby functioning in cell division and cell elongation (McCurdy et al., 2001; Havelková et al., 2015). The mutation of *ARPC2* in Arabidopsis (*Arabidopsis thaliana*) affected the shapes of a variety of cell types and reduced total shoot growth (El-Assal et al., 2004). Plants deficient in *ARP* expression show developmental phenotypes including defects in leaves, stems, and floral organs (Kandasamy et al., 2005). Arabidopsis plants lacking *ARPC2* expression show sharply reduced seed yield (Gachomo et al., 2014).

Gibberellic acids (GAs) are plant hormones with a wide range of developmental activities, including plant elongation (Kaufman, 1983; Harberd et al., 1998; Kende et al., 1998). GA-deficient plants were significantly shorter than the WT (Fridborg et al., 1999; Hedden and Phillips, 2000), but their height was restored to WT levels by applying GA3 (Lockhart, 1956). Recent progress has been made in identifying genes involved in GA metabolism and signaling pathways (Tuan et al., 2018). GA biosynthesis involves a series of pathways in which metabolic enzymes interact (Sakamoto et al., 2004). Copalyl diphosphate synthase (CPS), a terpene cyclase, catalyzes the first specific step of the GA biosynthesis pathway to produce *ent*-kaurene oxidase (Sun and Kamiya, 1994; Tudzynski et al., 1998; Sun, 2008). Morphology of *CPS* overexpressing-transgenic rice show similar to the WT (Sun and Kamiya, 1994), despite increased expression of the *ent*-kaurene gene (Fleet et al., 2003). Within the GA biosynthesis pathway, dioxygenases are important (Hedden and Phillips, 2000) for providing GA for various developmental processes (Rieu et al., 2008), and the total biosynthesis of bioactive GA is ultimately controlled by the dioxygenases GA3ox and GA20ox (Igielski and Kępczyńska, 2017). Different expression patterns of *GA20ox* contribute to phenotypic variation in Arabidopsis (Rieu et al., 2008), with *GA20ox* overexpression increasing the production of bioactive GAs, which influence plant growth (Huang et al., 1998). In rice, meanwhile, functionally deficient mutants of *GA20ox* show a typical dwarf phenotype (Barboza et al., 2013; Santoso et al., 2020).

In this current study, we used the Ac/Ds knockout system to generate new loss-of-function rice mutants with growth defects. By analyzing these mutants, we identified a novel gene encoding the ARPC2 subunit, which we named *gibberellic acid sensitive dwarf* (*GASD*). To investigate the role of *OsGASD* in plant development, we performed phenotypic and transcriptomic analysis of *OsGASD*-overexpressing (Ox) and *OsGASD* knockout (Ko) plants. Based on our findings, we propose that OsGASD is involved in the GA biosynthetic pathway and plays important roles in regulating plant growth and productivity. Our findings also shed light on the interactions between ARP genes and GAs.

## Materials and methods

### Plant materials and growth conditions

The rice cultivar *Oryza sativa* L. ‘Dongjin’ was used for all experiments including the establishment of Ac/Ds tagging system and screening of Ds insertion lines (Kolesnik et al., 2004). Rice plants were cultivated on MS media in a growth chamber (32°C, light/dark cycle of 16/8 h). After germination, plants were transferred to soil and grown in greenhouse. The daily high and low temperatures during the summer in the growing sites and in the greenhouse located at Dong-A University, Korea (128:96E/35:11N) were typically 32°C and 20°C, respectively. To construct *OsGASD* overexpression transgenic rice plants, the full length (1,035bp) of the *OsGASD* gene were inserted into *pENTR™*/*D-TOPO* (Invitrogen, Carlsbad, CA, USA). The recombination reaction between the entry and destination vectors was carried out using LR Clonase™ II enzyme mix (Invitrogen, Carlsbad, CA, USA) according to the manufacturer’s instructions. The destination vectors used *pH7WG2D.1* and introduced into *Agrobacterium tumefaciens* (EHA105) by electroporation. Generation of *OsGASD* transgenic plants followed a previously described method (Yang et al., 2018).

### Analysis of GA content measurement

At the 30 days after germination, 10g of culms and leaves of wild type plant, *OsGASD* overexpression plant and *osgasd* knockout mutant were grounded into powder blender with liquid N2 and then extracted with 80% methanol three times. The extract was evaporated in a rotation vacuum evaporator at 40°C to remove methanol and adjusted to pH 2.8-3.0 using HCl, and partitioned three times with equal volume of ethyl acetate. The combined substances dehydrate in vacuo at 42°C to remove ethyl acetate and then dissolved in 5mL 100% methanol, finally the solution was passed through chromatographed by HPLC (Hitachi, Chiyoda, Japan). The samples were chromatographed by HPLC on a C18 column (150 X 4.6 mm). Methanol - 2% acetic acid - water (40:40:20) solution passed column with the flow rate of 2mL/min and detecting wavelength of 254nm. Then 20uL of each sample was injected into the column and measured GA3 concentration.

### Histochemical analysis and cross section

To histochemical analysis since Ds line had a promoterless GUS for trapping the expression of Ds inserted endogenous gene, histochemical staining was performed to analyze of GUS expression (Jefferson et al., 1987). Different stages of leaves, panicles and roots were freshly collected from the mutants and wild type plants (as a negative control) and then treated with staining solution (0.02M 5-bromo-4-chloro-3-indolyl-bb-D-glucuronide, 0.1M NaH_2_PO_4_, 0.25M ethylenediaminetetraacetic acid (EDTA), 5mM potassium ferricyanide, 5mM potassium ferrocyanide, 1.0% (v:v) Triton X-100, pH 7.0) and incubated overnight at 37°C. Cross section of leaf blades sample was fixed in FAA and then analysis was performed as previously described method (Bae et al., 2021).

### Mutant genotyping and inverse PCR

The genomic DNA was isolated using the DNA extract Kit (MN, Dueren, Germany) from the leaves of wild-type and mutant. It was confirmed that the T-DNA was introduced into the rice knockout mutant using PCR amplification. To confirm of homozygous mutant, two gene specific primer P1, P2 and the Ds specific primer P3 were used. The amplification program consisted of an initial step at 94°C for 5 min, 35 cycles (30 sec at 94°C, 30 sec at 55°C, 1 min at 72°C), and a final step at 72°C for 10 min. To obtain the Ds flanking DNA sequences, inverse PCR (I-PCR) was performed using genomic DNA insolated and purified from the mutants and wild type plants (as a negative control). 1 µg genomic DNA was digested with *NlaIII* (10unit/ul (NEB, Ipswich, MA, USA)) at 37°C for 2.5hrs. Digested DNA was purified and self-ligated using T4 DNA ligase (NEB, Ipswich, MA, USA) at 16°C for overnight. The following primers used to do the I-PCR is d 3′ ends of Ds: Ds3-37, Ds3I-105, Ds3-4 and Ds3I-150. The amplification program comprised 2 step, 1st PCR is an initial step at 94°C for 3 min, 30 cycles (15 sec at 94°C, 1 min at 55°C, 2 min 30 sec at 72°C), and a final step at 72°C for 10 min. 2nd PCR is an initial step at 94°C for 3 min, 30 cycles (30 sec at 94°C, 30 sec at 55°C, 1 min at 72°C), and a final step at 72°C for 5 min.

### Reverse transcription quantitative polymerase chain reaction and transcriptome analysis

As previously reported, total RNA was isolated using the RNeasy Kit (Qiagen, Hilden, Germany) according to the manufacturer’s instructions for transcriptome analysis and quantitative real-time PCR (RT-qPCR) analysis. First-strand cDNAs were synthesized using 1 µg of total RNA with a cDNA Synthesis Kit (Takara, Kusatsu, Shiga, Japan), to serve as the templates for RT-qPCR. To determine levels of gene expression, RT-qPCR was performed and values were automatically calculated using a CFX94 Real-time PCR Detection System and CFX Manager software (Bio-Rad. Hercules, CA, USA) following a standard protocol. The sequences of primers used in RT-qPCR analysis are provided in **Supplementary Table S3**.

The total RNA was purified with NucleoSpin RNA clean-up Kit (MN, Dueren, Germany) and transcriptome analysis was performed with novaseq (Macrogen, Seoul, Korea). After trimming the low-data sequence reads to the reference *Oryza sativa japonica* (Rice Annotation Project Database). The transcripts were assembled and normalized by Fragment Per Kilobase of transcript per Killion mapped reads (FPKM), and the DEGs in each *OsGASD* Ox plant versus NT and *osgasd* Ko mutant vs NT were selected by |log2 (fold change) | ≥ 1 and |log2 (fold change)| ≤ −1 using a P value <0.05. The data set can be found at from SRA database with series accession number PRJNA867555 for RNA-sequencing data.

### Phylogenetic and grain yield analysis

The BLAST sequence, flanking DNA sequence and Ds insertion site analysis programs from NCBI (www.ncbi.nlm.nih.gov/) and Gramene web site (www.gramene.org/) were used to search for homologous gene. The multiple sequence alignments were performed with CLUSTAL W (http://www.genome.jp/tools/clustalw). Transgenic plants were generated and developed in GMO field located at Kyungpook National University, Korea (128:34 E/36:15N), and at the 40 days after ripening stage, 30 indicate independent lines of transgenic plants were harvested and investigated major agricultural traits such as panicle length, internode length, plant height, panicle length, number of panicle, number of filled grain, number of spikelet per panicle, filling rate, total grain weight, 1,000 grain weight, seed length and width compared with wild-type as a control.

### Accession number

The sequence data from this article can be found in the Rice Annotation Project (RAP-DB) under the following accession numbers: *GASD (Os04g0512300), CPS (Os02g0278700), KS (Os04g0611800), KO (Os06g0570100), KAO (Os06g0110000), GA20 (Os03g0856700), GA2 (Os05g0158600), GH3-9 (Os07g0576500), CCR5 (Os01g0283700), UFGT (Os06g0192100), KS8 (Os11g0474800), TEM1 (Os01g0141000), Grx_C7(Os01g0368900)*, and *Actin1 (Os03g0718100).*

## Results

### Isolation and characterization of a *osgasd* knock-out mutant

**Figure 1:**
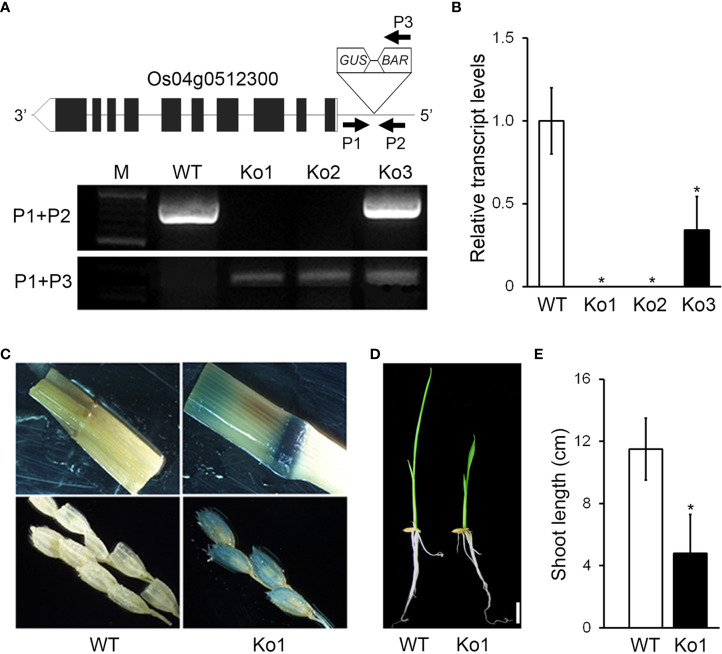
Identification and characterization of *osgasd* knockout mutant plants. **(A)** Diagram of *osgasd* knockout mutant (Ko) with the position of the inserted T-DNA (triangle) in the Os04g0512300 gene. Gene-specific (P1 and P2) and T-DNA left border (P3) primers used for PCR genotyping. WT indicates non-transgenic wild-type plants lacking a T-DNA insertion (PCR products amplified by only P1+P2). PCR products amplified with the indicated P1+P3 primer sets and both P1+P2 and P1+P3 were homozygous and heterozygous plants, respectively. M, DNA size marker. **(B)** RT-qPCR analysis showing transcript levels of *OsGASD* in the WT plant or *osgasd* mutants. Error bars indicate SD. OsActin1 (Os03g0718100) was used as an internal control and relative expression levels are shown in fold values. Asterisks indicate statistically significant differences between the corresponding samples and their control (p-value<0.1, Student’s t-test). Lines 1 (ko1) was used in this study. **(C)** Analysis of GUS staining in panicles neck node and spikelet of WT and *osgasd* knockout mutant which is carrying the P35S::GUS transgenes. **(D, E)** Comparison of shoot length in wild-type and *osgasd* knock mutant (Ko1). **(D)** Growth of wild-type plants and Ko1 mutants grown on MS media for 10 days (Scare bar = 1 cm).

To study the functions of genes involved in rice development, we generated T-DNA insertion mutants by Ac/Ds transposon insertion mutagenesis and selected a mutant with a dwarf phenotype. We then performed inverse PCR (see Materials and Methods for details) to identify the T-DNA insertion site in the mutant (**Figure 1**). Sequence analysis of the PCR products revealed that the DS element was located in the 5′UTR of the Os04g0512300 locus (**Figure 1A**). To select a homozygous mutant, we performed PCR using a Ds border primer (P3) paired with either a primer from near the 5′ end of the Ds element (P1) or one from near the 5′ end (P2). With the P1+P2 primers, we expected that genomic DNA from WT or heterozygous plants would to generate a PCR product, whereas genomic DNA from the mutants would not. In contrast, with the P1+P3 primers, the genomic DNA from mutants (both homozygote and heterozygote) was expected to generate a PCR product, whereas genomic DNA from the WT would not. Indeed, the *osgasd* homozygous mutants produced PCR products only using the P1+P3 primers, including the Ko mutants Ko1 and Ko2 (**Figure 1A**).

When we examined the transcript level of *OsGASD* (GenBank accession number KF741775) by RT-qPCR, both *osgasd* homozygous mutants (Ko1 and Ko2) showed suppressed expression of this gene compared to WT plants (**Figure 1B**). To confirm the T-DNA insertion in the mutants, we performed histochemical staining for GUS activity, as a *35S::GUS* cassette was transformed into the plants along with the DS element, and detected staining at panicle neck nodes and spikelets of the *osgasd* but not WT plants (**Figure 1C**). When we grew plants of both genotypes, the young shoots of the *osgasd* homozygous mutant were shorter than those of the WT. These results indicate that *OsGASD* is important for shoot growth in young plants.

To investigate the function of *OsGASD* in rice, we analyzed the complete deduced amino acid sequence of OsGASD by performing a Conserved Domain Search Service (CD Search) and BLAST analysis (**Figure S1**). The OsGASD protein is predicted to contained a p34-Arc subunit of the Actin-related protein 2/3 complex (Arp2/3). We also analyzed homologs of GASD and putative ARPC2s in other species. OsGASD shared approximately 15–35% sequence identity with its homologs from Arabidopsis [*Arabidopsis thaliana*], cattle [*Bos taurus*], fruit fly [*Drosophila melanogaster*], nematode [*Caenorhabditis elegans*], and yeast [*Saccharomyces cerevisiae*]). AtARPC2B and OsARPC2B share C-terminal amino acids representing a conserved domain of ARPC2Bs. The subcellular localizations of ARPs are in the nucleus to perform their specific function. We investigated the subcellular localization of fluorescent protein-tagged OsGASD and DAPI (4′,6-diamidino-2-phenylindole) that is nuclear staining dye using a rice protoplast transient expression system (**Figure S2**). OsGASD co‐localized with DAPI. This indicates that OsGASD is nucleus localizing proteins. These results indicate that *OsGASD* encodes a ARPC2B protein that is involved in plant development.

### *OsGASD* expression is response to GA-mediated plant growth

**Figure 2:**
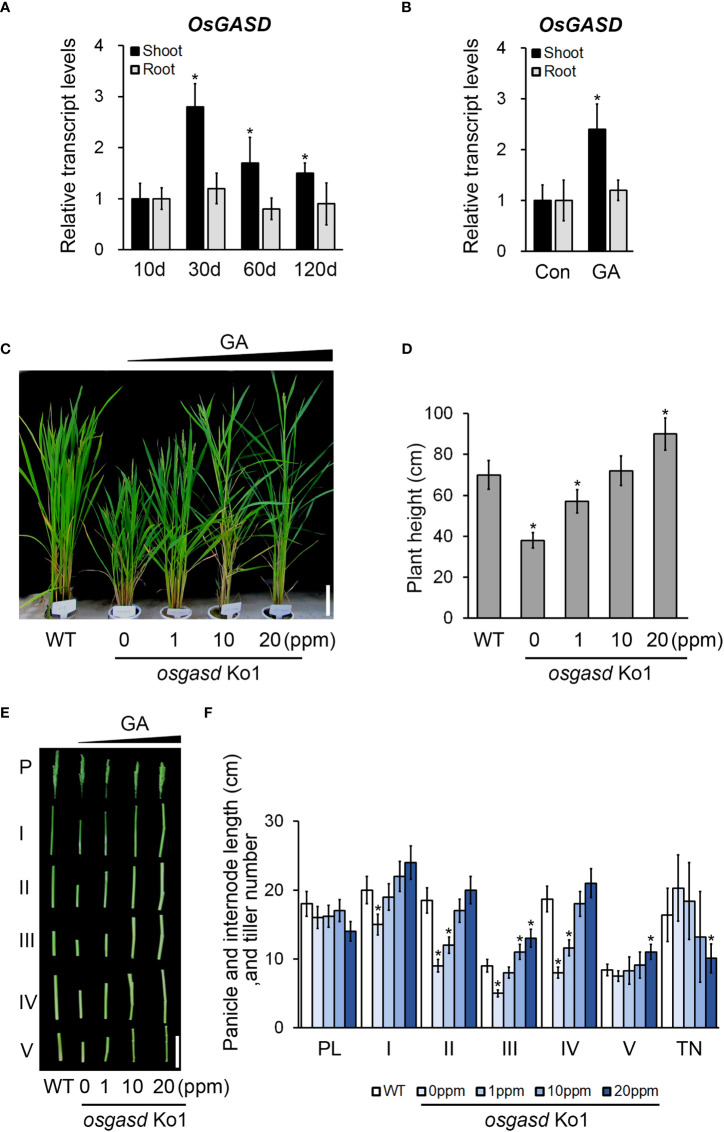
Analysis of *OsGASD* expression and effect of *osgasd* knockout mutant morphology in response to GA. **(A)** RT-qPCR analysis shows expression patterns of *OsGASD* in shoot and root. **(B)** The transcript level of *OsGASD* is shown in response to GA. Ten-day-old wild-type seedlings (Dongjin) were treated with 100 µM GA3 for 15min. Control plants were not treated with GA (Con). Error bars indicate SD. OsActin1 (Os03g0718100) was used as an internal control and relative expression levels are shown in fold values. Asterisks indicate statistically significant differences between the corresponding samples and their control (p-value<0.1, Student’s t-test). **(C-F)** Growth of *osgasd* knockout mutant grown on green house. (C and D) At the panicle heading stage (14 weeks) of *osgasd* knockout mutant treated with the indicated concentration of GA for 1, 10, 20 mg/L (ppm) **(C)** GA_3_ solution were sprayed with distilled water as control. Scale bar=15cm **(D)** Plant height measured seven days later for analysis. **(E, F)** P and PL indicated the panicle and panicle length. I to V indicated the corresponding internodes from top to bottom. Scale bar=10cm **(E)**. Quantification of plant height **(D)**, panicle and internodes length, and tiller number **(F)** in *osgasd* knockout mutant treated with GA (n>20). WT plants did not treat with GA. Error bars indicate SD. Asterisks indicate statistically significant differences between the corresponding samples and their control (p-value<0.1, Student’s t-test).

Since the *osgasd* Ko mutant had a dwarf phenotype beginning at an early stage, we suspected that the function of OsGASD is response to phytohormones, which function as growth regulators. We analyzed the expression patterns of *OsGASD* in these mutants in various tissues in response to Gibberellic acid (GA3) (**Figure 2** and **Figure S2**). The expression of *OsGASD* was higher in shoots than in roots in 30-, 60-, and 120-day-old rice (**Figure 2A**). Gibberellic acid (GA3) treatment increased the expression of *OsGASD* in shoots, but not in roots (**Figure 2B**). These results suggest that *OsGASD* is involved in GA response.

Therefore, we expected that the *osgasd* Ko mutant lacking *OsGASD* activity would exhibit some morphological changes in response to GA. We analyzed the sensitivity of these mutants and WT to GA treatment (**Figure S3** and **Figures 2C-F**). In response to GA spray treatment, 5-weeks-old *osgasd* Ko mutants increase of plant height compared to the WT (**Figures S3A, B**). The plant height of 14-week-old *osgasd* Ko mutants increased with increasing GA3 concentration, with the height of the *osgasd* Ko plants treated with 10 ppm GA3 being restored to level similar to those of WT plants (**Figures 2C, D**). We also analyzed internode elongation, panicle length, and tiller number in the *osgasd* Ko mutant (**Figures 2E, F**). With increasing GA3 concentration, the lengths of the first, second, third, fourth and fifth internodes in the mutant increased, while the panicle length was not significantly altered, and the tiller number decreased. These results, together with the expression pattern of *OsGASD* and the GA sensitivity of the *osgasd* Ko mutant, suggest that OsGASD function is related to GA biosynthesis during plant growth.

### *OsGASD* expression is involved in GA biosynthesis in plants

To explore the function of *OsGASD* in rice, we generated transgenic rice plants overexpressing this gene (*OsGASD* Ox; **Figure S3**) by *Agrobacterium*-mediated transformation of callus, followed by antibiotic selection. We then performed RT-PCR analysis to identify the transgenic lines (**Figure S3B**). Lines 2, 4, and 7 were selected for functional analysis of *OsGASD*. These transgenic plants showed increased plant height and internode length compared to WT plants (**Figures S3C-E**).

**Figure 3:**
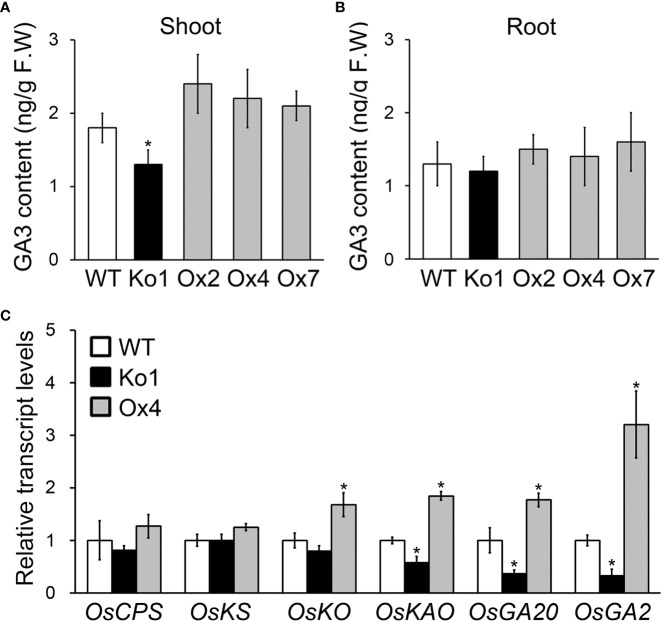
Analysis of GA3 concentrations and GA biosynthesis genes expression in *osgasd* knockout mutant and *OsGASD*-overexpressing transgenic plants. **(A, B)** GA3 concentrations were measured in the shoots **(A)** and roots **(B)** of Ko, Ox and WT plants grown on MS media for 30 days. **(C)** RT-qPCR analysis shows changes in transcriptional expression levels in shoot of Ko, Ox and WT plants. CPS, Copalyl diphosphate synthase; KS, ent-kaurene synthase; KO, ent- kaurene oxidase; KAO, ent- kaurenoic acid hydroxylase; GA20, GA 20 oxidase; GA2, GA 2 oxidase. Error bars indicate SD. OsActin1 (Os03g0718100) was used as an internal control and relative expression levels are shown in fold values. Asterisks indicate statistically significant differences between the corresponding samples and their control (p-value<0.1, Student’s t-test).

To investigate function of *OsGASD*, we analyzed the GA3 concentrations and expression of GA biosynthesis genes in *OsGASD* Ox, *osgasd* Ko mutant, and WT plants (**Figure 3**). The *OsGASD* Ox plants exhibited increased GA3 content in shoots, whereas the *osgasd* Ko mutant showed decreased GA3 content in these organs compared to WT plants (**Figure 3A**). However, the GA3 contents in the roots of *OsGASD* Ox and *osgasd* Ko plants did not significantly differ from those of the WT (**Figure 3B**), indicating that *OsGASD* is related to GA biosynthesis in shoots.

If *OsGASD* is involved in GA biosynthesis in shoots, we hypothesized that OsGASD affects the expression of GA biosynthesis gene, such as the genes encoding copalyl diphosphate synthase (*CPS*), *ent*-kaurene synthase (*KS*), *ent*-kaurenoic acid hydroxylase (*KAO*), GA 20 oxidase (*GA20*), and GA 2 oxidase (*GA2*). To address this issue, we analyzed the transcript levels of these GA biosynthesis genes in the shoots of young (10-day-old) *OsGASD* Ox and *osgasd* Ko plants (**Figure 3C**). *KAO*, *GA20* and *GA2* transcript levels were higher in *OsGASD* Ox plants than in WT plants. By contrast, *KAO*, *GA20* and *GA2* transcript levels were lower in *osgasd* Ko than in WT plants. These results point to an association between *OsGASD* expression and GA biosynthesis in plants.

### Overexpression of *OsGASD* enhances plant growth and grain yield

**Figure 4:**
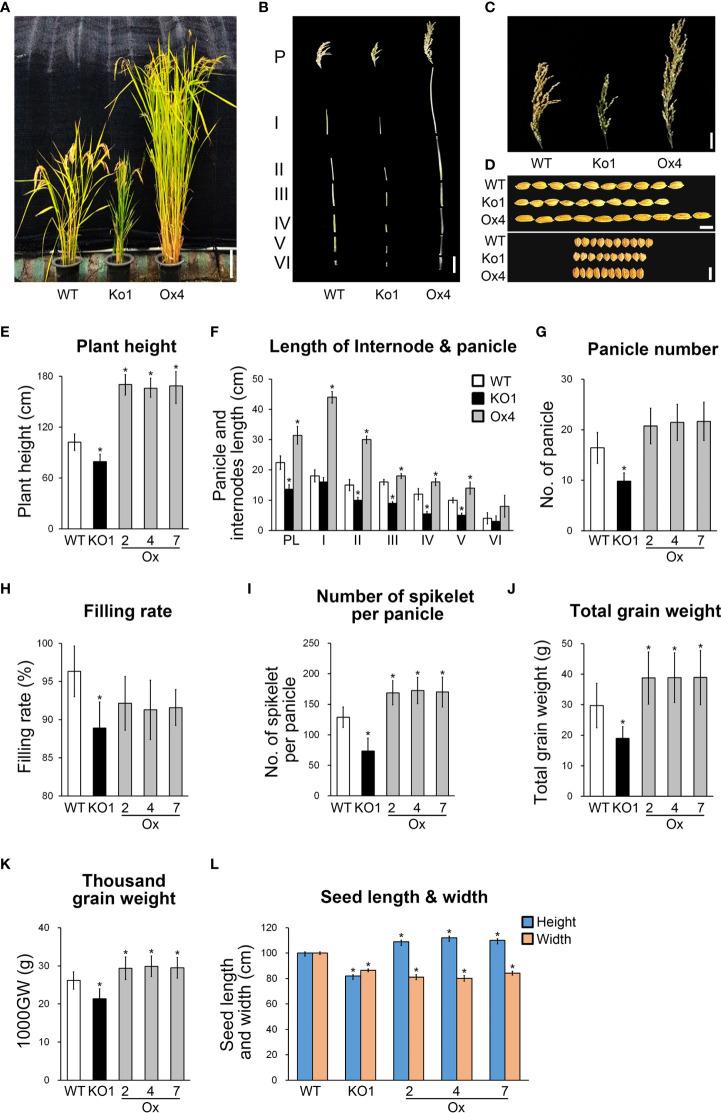
Expression of *OsGASD* affects agronomic traits under normal growth conditions. **(A)** Morphology of 25-week-old wild-type (WT), osgasd knockout mutant (Ko) and OsGASD-overexpressing (Ox) plants grown in the field. Scale bar = 15 cm **(B)** Images show the main panicles and internodes. P and PL indicated the panicle and panicle length. I to V indicated the corresponding internodes from top to bottom. Scale bar=10cm. **(C)** Images show the panicles with grains. The panicles shown were grain from a single main panicle. **(D)** Images show the size of the grains Ox and Ko indicate OsGASD-overexpressing and mutant plants, respectively. WT indicates wild-type plants. Scale bars = 15 cm in **(A)** 5cm in **(B)**, 1cm in 0.2cm in **(D)**. **(E–K)** The analysis of agronomic traits of the Ox and Ko plants grown in field. Plant height **(E)**, internode length and panicle **(F)**, panicle number **(G)**, filling rate **(H)**, number of spikelet per panicle **(I)**, total grain weight **(J)** and thousand grain weight **(K)**. **(L)** analysis of seed length and width of the Ox and Ko plants. Three independent lines of T4 OsGASD-Ox and -Ko plants were analyzed together with their wild-type counterparts grown in the same conditions. The values represent the percentage of the mean values (n = 30) listed in **Tables S1**. Mean values of WT controls were set at 100% as a reference. Asterisks indicate statistically significant differences between the corresponding samples and their control (p-value<0.1, Student’s t-test).

Since GAs are phytohormones that affect plant productivity, we expected that the expression of *OsGASD* would also affect rice productivity. Analysis of the morphology of whole plants, internodes, panicles, and grains from *OsGASD* Ox, *osgasd* Ko mutant, and WT plants revealed that *OsGASD* Ox showed increased plant and grain size, including increased plant height, internode length, panicle number, and grain weight, whereas the *osgasd* Ko mutants exhibited reduced plant and grain size (**Figures 4A-F, L**; **Table S1**). To further explore rice productivity, we analyzed agronomic traits including the number of panicles, filling rate, number of spikelets per panicle, total grain weight, and 1,000-grain weight (**Figures 4G-K**; **Table S1**). The number of panicles was approximately 40% lower in the *osgasd* Ko mutants compared to the WT plants. However, the number of panicles was not significantly elevated in *OsGASD* Ox plants. Total grain weight, the number of filled grains, and 1,000-grain weight of *OsGASD* Ox plants increased by approximately 27%, 30% and 13%, respectively, compared to WT plants. However, the filling rate was reduced in *OsGASD* Ox plants compared to WT plants. By contrast, the *osgasd* Ko mutants showed reduced values for all parameters compared to WT plants. Consequently, the total grain weight and total number of filled grains were higher in *OsGASD* Ox plants, but lower in *osgasd* Ko plants, compared to WT plants. Furthermore, the seed size and 1,000-grain weight differed among lines (**Figures 4K, L**). These results suggest that the increased grain yield in *OsGASD* Ox is largely due to increases in panicle length, number of filled grains, and grain weight, supporting the notion that the expression of *OsGASD* affects the development of seeds that form in the panicle.

### *OsGASD* expression is involved in flag leaf development

**Figure 5:**
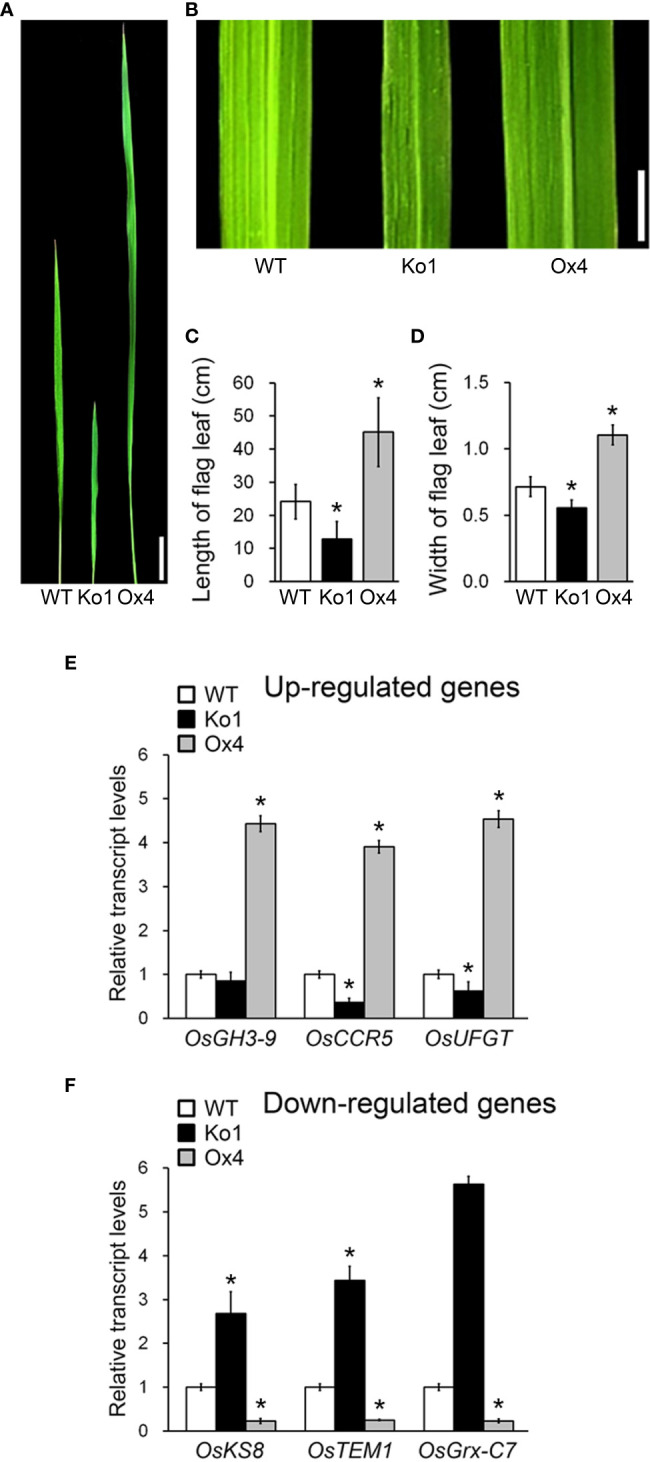
Analysis of flag leaf morphology and plant development related transcript level in *osgasd* knockout mutant and *OsGASD*-overexpressing transgenic plants. **(A, B)** Morphology of flag leaf in wild-type (WT), *osgasd* knockout mutant (Ko) and *OsGASD*-overexpressing (Ox) plants grown in the field. Image show flag leaf **(A)** and flag leaf surface **(B)**. Scale bar = 1cm **(A)** and 0.5cm **(B)**. **(C, D)** Length ^©^ and width **(D)** of flag leaf in WT, *osgasd* Ko and *OsGASD-*Ox plants. Error bars indicate SD (n = 20). **(E, F)** Analysis of plant development related genes expression in WT, Ko and Ox plants. The transcripts were selected by transcriptome analysis in flag leaf of *osgasd* Ko, *OsGASD-*Ox and WT plants. RT-qPCR analysis shows up-regulated **(E)** and down-regulated **(F)** transcriptional expression levels. The gene name and accession liseted in **Tables S2**. Error bars indicate SD. OsActin1 (Os03g0718100) was used as an internal control and relative expression levels are shown in fold values. Asterisks indicate statistically significant differences between the corresponding samples and their control (p-value<0.1, Student’s t-test).

Flag leaf development affects rice productivity (Saitoh et al., 2002; Acevedo-Siaca et al., 2021; Makino et al., 2022). If OsGASD is involved regulating rice productivity by influencing flag leaf development, we reasoned that altering *OsGASD* expression would affect the morphology of flag leaves. To investigate the role of *OsGASD* in flag leaf development, we analyzed the phenotypes of *OsGASD* Ox, *osgasd* Ko, and WT plants (**Figures 5A-D**). In 25-week-old plants, the length and width of flag leaves were both increased to approximately 2-fold in *OsGASD* Ox plants compared to WT plants, whereas *osgasd* Ko flag leaves were smaller than those of WT plants.

To further explore these effects, we analyzed the transcriptomes of flag leaves from *OsGASD* Ox, *osgasd* Ko, and WT plants at the mature stage (25-week-old plants) by transcriptome sequencing (RNA-seq) analysis. An analysis of differentially expressed genes (DEGs) indicated that 46 genes were upregulated in *OsGASD* Ox and downregulated in *osgasd* Ko compared to WT plants. Moreover, 122 genes were downregulated in *OsGASD* Ox and upregulated in *osgasd* Ko plants. To identify key genes, we examined the putative functions of the DEGs by Gene Ontology (GO) enrichment analysis, finding that they included a number of genes related to plant development. We selected genes with previously reported functions in rice or homologous genes reported in Arabidopsis (**Table S2**). A previous study had indicated that plants overexpressing *OsGH3-9*, *OsCCR5*, and *OsUFGT* showed enhanced shoot growth and seed formation due to increased cell development and elongation (Nakazawa et al., 2001; Takase et al., 2004; Thévenin et al., 2011; Dong et al., 2020). *OsKS8*-, *OsTEM1*-, and *OsGrx-C7*-overexpressing plants showed phenotypes similar to those of the *osgasd* Ko mutants, such as a dwarf stature and poor plant growth (Fleet et al., 2003; Hu et al., 2004; El-Kereamy et al., 2015). To validated the expression patterns of these selected genes, we analyzed their expression patterns by RT-qPCR, obtaining results similar to the RNA-seq results (**Figures 5E, F**). *OsCCR5*, *OsNCED3* and *OsUFGT* were upregulated in *OsGASD* Ox and downregulated in *osgasd* Ko. However, *OsGH3-9* was upregulated in *OsGASD* Ox but was not downregulated in *osgasd* Ko (**Figure 5E**). *OsKS8, OsTEM1* and *OsGrx-C7* were downregulated in *OsGASD* Ox and upregulated in *osgasd* Ko (**Figure 5F**). These results indicate that OsGASD regulates the expression of plant development-related genes involved in flag leaf development and plant productivity, suggesting that it is helps control overall plant growth by regulating *OsGASD*-mediated GA biosynthesis.

## Discussion

ARP2/3 complex subunits are involved in shaping walled cells and branch structure in plants (Wasteneys and Galway, 2003; Kandasamy et al., 2005). For example, a knockout mutant of *DISTORTED2 (DIS2)*, which has ARPC2 activity, has distorted trichomes (El-Din El-Assal et al., 2004). Here we identified the *OsGASD* gene, which encodes a homolog of ARPC2 in rice. In *osgasd* Ko lines, shoot growth was reduced, leading to a dwarf phenotype in plants during all stages of development. *OsGASD* was expressed at higher levels in shoots than in roots (**Figures 1D, E**, **2A**, **4A,E**). Furthermore, grain size and total grain weight were significantly higher in *OsGASD* Ox transgenic rice than in WT plants but lower in *osgasd* Ko mutants (**Figure 4**). Finally, the length and width of flag leaves were higher in *OsGASD* Ox plants compared to WT plants. Together, these results indicate that *OsGASD* is involved in shoot and seed development.

GAs are closely related to plant growth processes, such as seed and flower development, leaf expansion, and shoot elongation. We therefore analyzed *OsGASD* expression in response to GA, finding that it increased in shoots (but not roots) in response to GA treatment (**Figure 2**, **S2**). In addition, plant height and the lengths of panicles and internodes in the *osgasd* Ko mutant were restored to WT levels in response to GA treatment (**Figures 2B-F**). Furthermore, the shoots of *OsGASD* Ox plants had higher GA3 contents than WT shoots, whereas the shoots of the *osgasd* Ko mutant had lower GA3 content than WT shoots. However, GA3 contents in the roots of both *OsGASD* Ox and *osgasd* Ko plants were similar to those of WT plants (**Figures 3A, B**). These results suggest that *OsGASD* is involved in GA biosynthesis in rice shoots. In Arabidopsis, both *AtCPS*- and *AtCPS/AtKS*-overexpressing transgenic plants showed increased accumulation of *ent*-kaurene and *ent*-kaurenoic acid, two early intermediates in the GA biosynthetic pathway, but normal levels of bioactive GA (Fleet et al., 2003). Here, we analyzed the expression of key genes involved in GA biosynthesis, such *CPS*, *KS*, *KO*, *KAO*, *GA20*, and *GA2*. Whereas *KO*, *KAO*, *GA20*, and *GA2* were upregulated in the shoots of young *OsGASD* Ox plants, the expression of *CPS* and *KS* was not significantly altered (**Figure 3C**). These results indicate that OsGASD mediates the accumulation of bioactive GA in young shoots during development.

Moreover, we analyzed the transcriptomes of *OsGASD* Ox and *osgasd* Ko flag leaves by RNA-seq to explore the relationship between *OsGASD* expression and yield. The transcript levels of cell-development- and elongation-related genes were altered in these plants compared to the WT. Specifically, *OsGH3-9*, *OsCCR5*, and *OsUFGT* were upregulated in *OsGASD* Ox plants, whereas *OsKS8*, *OsTEM1* and *OsGrx-C7* were downregulated (**Figures 5E, F**). Together, these findings suggest that the *OsGASD* gene is important for GA biosynthesis and the development of rice shoots.

Previous studies have reported that plant growth processes, including cell elongation, were affected by the overexpression of the auxin-responsive *GH3* gene (Nakazawa et al., 2001), and its loss of function led to increased hypocotyl elongation (Takase et al., 2004). The cinnamoyl CoA reductase (*CCR*) is important role in lignin systhesis, which is involed in cell wall development and stress defense system in plant.suppression or absence of c*CCR* in Arabidopsis resulted in dwarfism and decreased floral organ formation (Thévenin et al., 2011, and Cui et al., 2022). Transgenic rice overexpressing *GSA1*, encoding a UDP-glucosyltransferase (UFGT), showed larger grain size than WT plants (Dong et al., 2020). The overexpression of *AtCPS* and *AtKS* increased bioactive GA contents and limited plant growth and development (Fleet et al., 2003). Overexpression of *RAV1* negatively affected leaf structure, indicating that altering the expression of this gene changes leaf structure (Hu et al., 2004). Transgenic rice plants overexpressing the glutaredoxin gene *GRX6* showed reduced grain yield and plant height but significantly increased grain size and weight (El-Kereamy et al., 2015).

However, it is not yet understood how the ARPC2 subunit OsGASD regulates the expression GA biosynthesis genes and cell-development-related genes. Based on the current findings, we propose that rice OsGASD regulates shoot development and grain size by regulating the expression of GA biosynthesis genes, thereby conferring high productivity.

## Data availability statement

The datasets presented in this study can be found in online repositories. The names of the repository/repositories and accession number(s) can be found in the article/**Supplementary Material**.

## Author contributions

TU, SH, WY, DK designed the experiments and wrote the manuscript. SH, WY and TU performed most of the experiments. JH and B-SC, PB performed part of the experiments. YC, SL, SM and KJ discussed and commented on the results and the manuscripts. All authors read and discussed the manuscript. WY and DK provided funding for research work as corresponding author. All authors contributed to the article and approved the submitted version.

## Supplementary material

Supplementary Figure 1 Analysis of amino acid between *OsGASD* and ARPC2s of other species. The red shade indicates conserved C-terminal domain of ARPC2B in Arabidopsis and rice. The underline indicates C-terminal alpha-helix of 43 residues of ARPC2s that is interacted with *ARPC4* (p20). *OsGASD* (KF741775), *AtDIS2* (BT005308), *AtARPC2B* (NM_179877), *OsARPC2A* (AP003709), *OsARPC2B* (CAE03390), B. taurus *BtARPC2* (1K8K_D), D. melanogaster *DmARPC2* (NP_610033), C. elegans *CeARPC2* (NP_741088) and S. cerevisiae ScARPC2 (NP_014433)

Supplementary Figure 2 Subcellular localization of OsGASD. Transient expression of 35S-GFP and 35S-OsGASD-GFP fusions construct in protoplast were carried out to determine the subcellular localization. Scale bars indicate 10 µm.

Supplementary Figure 3 Analysis of *osgasd* knockout mutant and WT in response to GA. **(A, B)** Growth of *osgasd* mutant and WT plants incubated on soil for 5-weeks-old (bar=10cm). WT **(A, C)** and *osgad* mutant **(B, D)** were sprayed once 5 days with 1, 10, 20 mg/L of GA3 or distilled water (0 ppm) or control plant were not treated with GA and water (Con). **(C, D)** All treated plants were measured height of plant. Each treated group had 15 plants and experiments were repeated three times. Asterisks indicate statistically significant differences between the corresponding samples and their control (p-value<0.1, Student’s t-test).

Supplementary Figure 4 Generation of transgenic rice plants overexpressing *OsGASD* gene. **(A)** Structure of the construct is show. LB, left border; RB, right border, HPT, hygromycin phosphotransferase. Northern blot analysis **(B)** of the *OsGASD* gene in the transgenic rice. Red boxes mark lines 2, 4 and 7 which were used in this study. **(C-E)** Analysis of overexpression of *OsGASD* plant (Ox) and *osgasd* knockout mutant (Ko1) phenotypes. **(C)** Morphology of 10-day-old wild-type (WT), overexpression of *OsGASD* plant (Ox) and *osgasd* knockout mutant (Ko1). Comparison of plant height **(D)** and internode length **(E)** were measured of the WT, Ox plants and Ko1 mutant. Error bars indicate SD. Asterisks indicate statistically significant differences between the corresponding samples and their control (p-value<0.1, Student’s t-test). Scale bar=1cm
