# Supplementary material for: Gibberellic acid sensitive dwarf encodes an ARPC2 subunit that mediates gibberellic acid biosynthesis, effects to grain yield in rice
Source: Front Plant Sci. 2022 Dec 22;13:1027688. doi: 10.3389/fpls.2022.1027688 (PMC9813395; doi:10.3389/fpls.2022.1027688)
Supplement: Supplementary file 1 [file DataSheet_1.docx]

Supplementary Material

# Supplementary Data

Supplementary Material should be uploaded separately on submission. Please include any supplementary data, figures and/or tables. All supplementary files are deposited to FigShare for permanent storage and receive a DOI.

Supplementary material is not typeset so please ensure that all information is clearly presented, the appropriate caption is included in the file and not in the manuscript, and that the style conforms to the rest of the article. To avoid discrepancies between the published article and the supplementary material, please do not add the title, author list, affiliations or correspondence in the supplementary files.

# Supplementary Figures and Tables

For more information on Supplementary Material and for details on the different file types accepted, please see [here](http://home.frontiersin.org/about/author-guidelines#SupplementaryMaterial). Figures, tables, and images will be published under a Creative Commons CC-BY licence and permission must be obtained for use of copyrighted material from other sources (including re-published/adapted/modified/partial figures and images from the internet). It is the responsibility of the authors to acquire the licenses, to follow any citation instructions requested by third-party rights holders, and cover any supplementary charges.

## Supplementary Figures

**Supplementary Figure 1.** ***Il6* expression in the developing and adult pituitary.** Gene expression levels of *Il6* in the pituitary (embryonic) or AL (neonatal, adult) at indicated ages. Graph shows mean ± SEM (n = 2-3, all individually shown; one-way ANOVA with Sidak’s multiple comparisons test, **P < 0.01, ***P < 0.001).

**Supplementary Figure 2. Expression of IL-6 and related cytokines in the IL-6 KO and damaged pituitary.** Proportion of GH^+^ cells in total AL cell population from DMG-WT and DMG-KO mice at 5 mo. Bars depict mean ± SEM (n = 3-4; unpaired t-test) (A). Gene expression levels of indicated genes in *Il6^-/-^* relative to *Il6^+/+^* AL. Bars depict mean ± SEM (n = 4, all individually shown; one-way ANOVA with Sidak’s multiple comparisons test, *P < 0.05, ****P < 0.0001) (B). *Il6* expression levels in DMG-WT AL at d4 and d11, relative to CTRL-WT (dotted line). Graph shows mean ± SEM (n = 3, all individually shown; 2way ANOVA with Sidak’s multiple comparisons test, **P < 0.01, ****P < 0.0001) (C). Gene expression levels of indicated genes in DMG-WT and DMG-KO AL at d4 and d11, relative to CTRL-WT and CTRL-KO (dotted line), respectively. Graphs show mean ± SEM (n = 3-5, all individually shown; 2way ANOVA with Tukey’s multiple comparisons test, *P < 0.05, **P < 0.01, ***P < 0.001, ****P < 0.0001; ns, non-significant). ND, not detected. *Cntf*, ciliary neurotrophic factor; *Ct1*, cardiotrophin-1; *Osm*, oncostatin M; *Lif*, leukemia-inhibitory factor; *Ifng*, interferon-γ; *Tnf*, tumor-necrosis factor (D).

## Supplementary Tables

**Supplementary Table 1. Overview of primary and secondary antibodies used for immunofluorescence staining**

**Supplementary Table 2. Overview of primer sequences used for RT-qPCR gene expression analysis**
