# Supplementary material for: Gibberellic acid sensitive dwarf encodes an ARPC2 subunit that mediates gibberellic acid biosynthesis, effects to grain yield in rice
Source: Front Plant Sci. 2022 Dec 22;13:1027688. doi: 10.3389/fpls.2022.1027688 (PMC9813395; doi:10.3389/fpls.2022.1027688)
Supplement: Supplementary file 2 [file Table_1.docx]

**SUPPLEMENTARY MATERIALS_TABLES**

**Table S1. Agronomic traits of T_4_ generation of *OsGASD*-overexpressing and -knockout plants under normal conditions.** Each parameter value represents the mean ± SD (n = 30). Ox indicate independent lines at T generation of *OsGASD*-overexpressing plants, and Ko1 indicates *osgasd* mutants. The percentage differences (%△) show increase or decrease in the parameters compared with their corresponding controls.

| **Constructs** | **Plant height (cm)** | **panicle**  **Length (cm)** | **No. of panicle** | **No. of filled grains** | **Number of spikelets per panicle** | **Filling rate** | **Total grain weight** | **1,000 grain weight.** |
| --- | --- | --- | --- | --- | --- | --- | --- | --- |
| **WT (dongjin)** | 102.36±1.73 | 22.40±1.52 | 16.40±3.05 | 123.62±7.15 | 128.83±16.68 | 96.33±2.33 | 29.71±7.28 | 26.17±0.28 |
| **Ko1** | 79.31±1.58 | 11.60±1.55 | 9.80±1.64 | 64.29±7.03 | 73.33±21.09 | 88.89±4.42 | 18.93±3.87 | 21.37±2.60 |
| %△ | -22.52 | -48.21 | -40.24 | -47.99 | -43.08 | -7.72 | -36.28 | -18.34 |
| **Ox 2** | 165.86±1.58 | 31.45±3.12 | 20.72±3.52 | 155.55±10.39 | 168.82±31.19 | 91.28±3.52 | 38.93±8.87 | 29.37±2.94 |
| %△ | 62.04 | 40.40 | 26.34 | 25.83 | 31.04 | -5.24 | 31.03 | 12.23 |
| **Ox 4** | 170.38±2.58 | 30.12±3.04 | 21.44±3.56 | 158.92±7.19 | 172.48±21.58 | 92.14±3.88 | 38.75±8.54 | 29.88±2.66 |
| %△ | 66.45 | 34.46 | 30.73 | 28.56 | 33.88 | -4.35 | 30.43 | 14.18 |
| **Ox 7** | 168.72±2.88 | 32.31±3.18 | 21.66±3.76 | 155.79±9.41 | 170.12±28.22 | 91.58±4.12 | 38.88±8.14 | 29.48±2.72 |
| %△ | 64.83 | 44.24 | 32.07 | 26.02 | 32.05 | -4.93 | 30.87 | 12.65 |

**Table S2. Significantly differentially expressed genes involved in cell elongation and development in *osgasd* knockout mutant and OsGASD-overexpressing transgenic plants.** Transcriptome analysis of flag leaf in wild-type (WT), *osgasd* knockout mutant (Ko) and *OsGASD*-overexpressing (Ox) plants grown in the field. Total RNA was prepared from flag leaf of 24-week-old plants. Mapping of sequence reads to the reference *Oryza sativa japonica* (Rice Annotation Project Database).

| **Accession No.** | **Name** | **Description** | **Log^2^ fold change** | | **p-value** |
| --- | --- | --- | --- | --- | --- |
|  |  |  | **WT vs Ox4** | **WT vs Ko1** |  |
| **Up-regulated genes** | |  |  |  |  |
| Os07g0576500 | *OsGH3-9* | Auxin-responsive GH3-8 protein | 3.14 | -2.28 | 0.05 |
| Os08g0167800 | *OsTPS30* | Sesquiterpene synthase | 2.68 | -2.36 | 0.00 |
| Os12g0125800 | *OsMATE52* | Transparent testa 12 protein | 2.29 | -1.47 | 0.04 |
| Os04g0607600 | *OsHKT7* | High-affinity K+ transporter (HKT) family protein | 2.20 | -1.44 | 0.01 |
| Os01g0283700 | *OsCCR5* | Cinnamoyl-CoA reductase | 1.70 | -3.45 | 0.00 |
| Os03g0645900 | *OsNCED3* | 9-cis-epoxycarotenoid dioxygenase | 1.35 | -1.34 | 0.00 |
| Os06g0192100 | *OsUFGT* | UDP-glucose flavonoid-3-O-glucosyltransferase | 1.15 | -3.00 | 0.00 |
| **Down regulated genes** | |  |  |  |  |
| Os09g0467200 | *OsGSTU17* | Response to abiotic stresses | -1.10 | 1.41 | 0.00 |
| Os11g0474800 | *OsKS8* | ent-kaurene synthase8, | -1.11 | 1.69 | 0.00 |
| Os01g0141000 | *OsTEM1* | Repression of photoperiodic flowering | -1.18 | 1.87 | 0.00 |
| Os02g0568700 | *OsKSL12* | Ent-kaurene synthase (KS) like diterpene synthase | -1.92 | 1.71 | 0.01 |
| Os01g0368900 | *OsGrx_C7* | Arsenic (As) stress response | -3.01 | 3.43 | 0.00 |
| Os12g0491800 | *OsKSL10* | Ent-kaurene synthase 1A | -3.28 | 1.04 | 0.01 |
| Os11g0666200 | *OsRLCK345* | Receptor-like Cytoplasmic Kinase | -4.24 | 2.43 | 0.00 |

**Table S3. Primer lists for our studies**

| Name | Forward primer (5'to3') | Reverse primer (5'to3') |
| --- | --- | --- |
| P1 | CCGCTTTCACTGGCAAAGA |  |
| P2 | CAAGGATGTGCTAGAGAGATC |  |
| P3 | CGGGATATACAAAACGGTAAACGG |  |
| DS3-4 | GTTACCGACCGTTTTCATCC |  |
| DS3-37 | TATGAAAATGAAAACGGTAGAGG |  |
| DS3I-105 | AAACGAACGGGATAAATACGG |  |
| DS3I-150 | GGTTAAAGTCGAAATCGGACG |  |
| OsGASD-OX | CACCATGCCTGTTGATCATACCTT | TCACTCCAGCTTCTGGTACT |
| OsActin1-qRT | GAACTGGTATGGTCAAGGCTG | ACACGGAGCTCGTTGTAGAAG |
| OsGASD-qRT | CCTGCTCAATTTCCAGTCCTAC | CATGATCTCCAGACGCTTTCTC |
| OsCPS-qRT | GCGTGCATTTTCGAACCAA | TTGGCCAGCACTGACACTCT |
| OsKS-qRT | GGGCGTCTCCTGAATGACA | CAGTGAGACACTGTTCAGCTTTCC |
| OsKO-qRT | TGCTACCAGCGACTATTGTGATTT | GTGCAGAAGTACCCAACATGCTT |
| OsKAO-qRT | CAGCAACGCAGAACGGATTAA | ACGTTGACGCAGCGAAGTG |
| OsGA20-qRT | GCCACTACAGGGCCGACAT | TGGTTGCAGGTGACGATGAT |
| OsGA2-qRT | TGACGATGATGACAGCGACAA | CCATAGGCATCGTCTGCAATT |
| OsGH3-9-qRT | TCACCGAGTTCCTCACCA | AGTTTCAAGACAGGTCCAATGA |
| OsCCR5-qRT | GCTGTCCATATGAACCCTAACA | TCGCGTAGCAATACCAGTTC |
| OsUFGT-qRT | AGTTGCAGGCGAAGGTG | GCACAGATTATCTCGACGAACT |
| OsKS8-qRT | TACAACACGACCAAGCGTATC | CTCACCATCACCTGCCATT |
| OsTEM1-qRT | GTGGAGCCGATTCGTGAG | GCGTTGTTCTTCTTGCAGTC |
| OsGrX_C7-qRT | ATGGACAGGGTGAACAGG | CCGAGCTCGCAGAAGAG |
