# Supplementary figures and images for: Gibberellic acid sensitive dwarf encodes an ARPC2 subunit that mediates gibberellic acid biosynthesis, effects to grain yield in rice
Source: Front Plant Sci. 2022 Dec 22;13:1027688. doi: 10.3389/fpls.2022.1027688 (PMC9813395; doi:10.3389/fpls.2022.1027688)

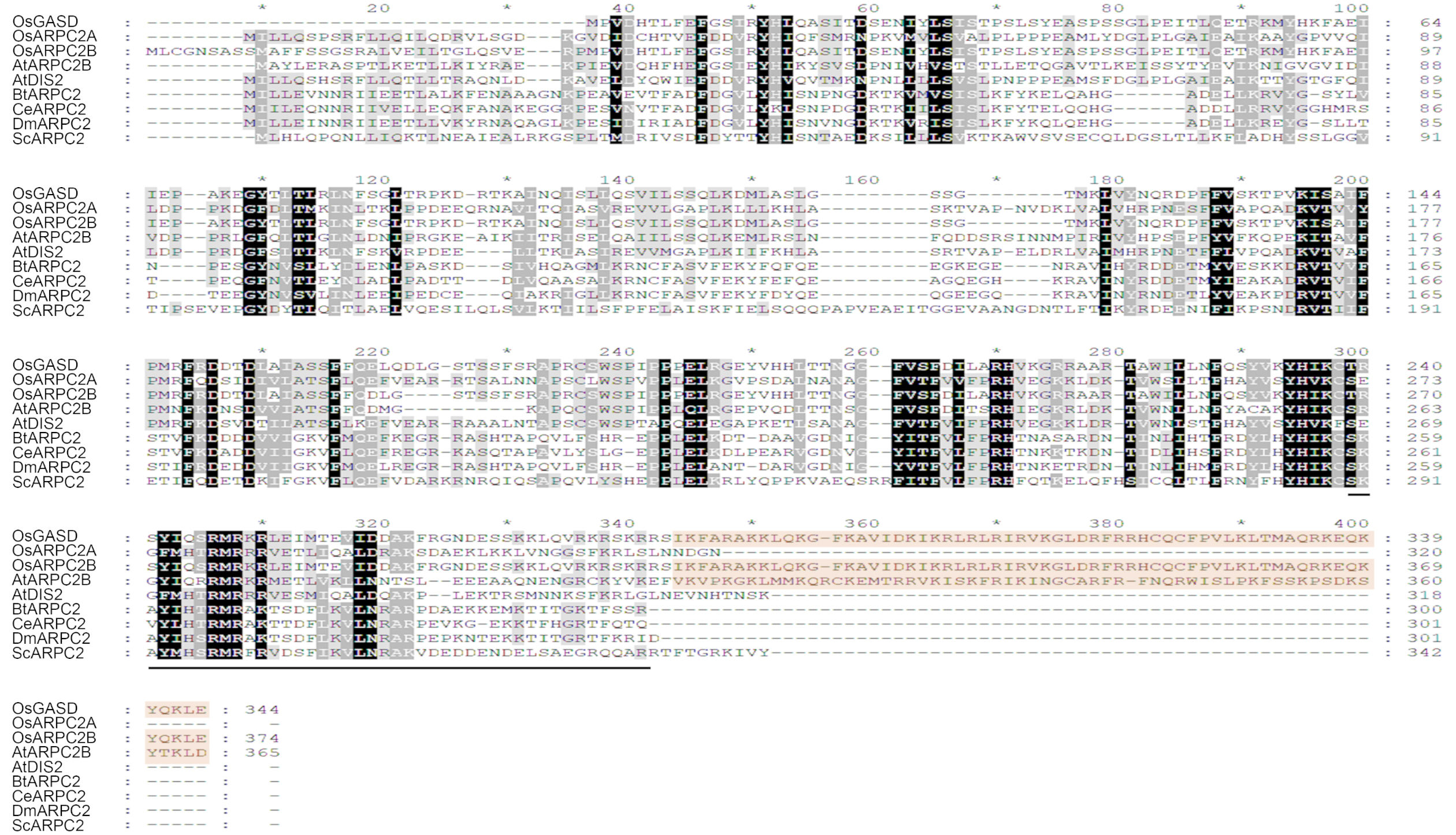

Supplement: Supplementary Figure 1 — Analysis of amino acid between OsGASD and ARPC2s of other species. The red shade indicates conserved C-terminal domain of ARPC2B in Arabidopsis and rice. The underline indicates C-terminal alpha-helix of 43 residues of ARPC2s that is interacted with ARPC4 (p20). OsGASD (KF741775), AtDIS2 (BT005308), AtARPC2B (NM_179877), OsARPC2A (AP003709), OsARPC2B (CAE03390), B. taurus BtARPC2 (1K8K_D), D. melanogaster DmARPC2 (NP_610033), C. elegans CeARPC2 (NP_741088) and S. cerevisiae ScARPC2 (NP_014433) [file Image_1.jpeg]

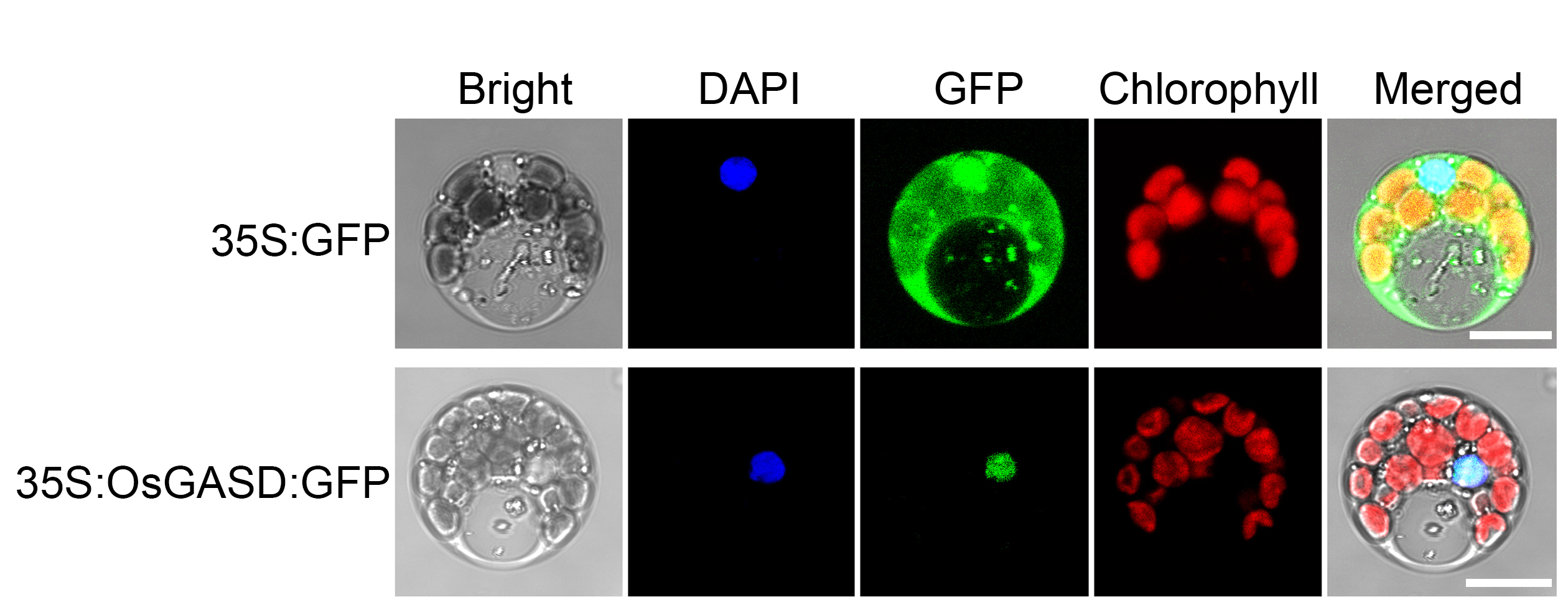

Supplement: Supplementary Figure 2 — Subcellular localization of OsGASD. Transient expression of 35S-GFP and 35S-OsGASD-GFP fusions construct in protoplast were carried out to determine the subcellular localization. Scale bars indicate 10 µm. [file Image_2.jpeg]

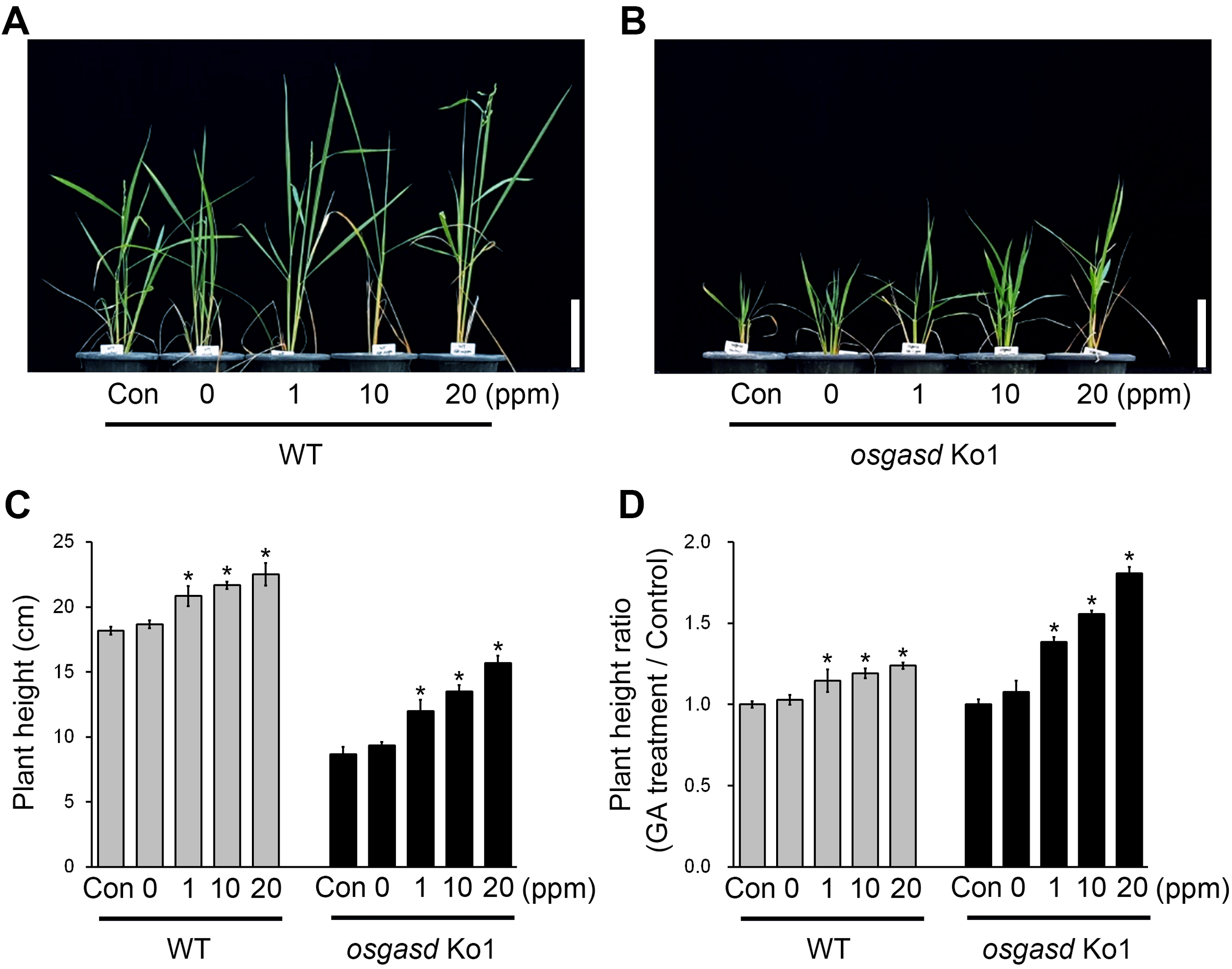

Supplement: Supplementary Figure 3 — Analysis of osgasd knockout mutant and WT in response to GA. (A, B) Growth of osgasd mutant and WT plants incubated on soil for 5-weeks-old (bar=10cm). WT (A, C) and osgad mutant (B, D) were sprayed once 5 days with 1, 10, 20 mg/L of GA3 or distilled water (0 ppm) or control plant were not treated with GA and water (Con). (C, D) All treated plants were measured height of plant. Each treated group had 15 plants and experiments were repeated three times. Asterisks indicate statistically significant differences between the corresponding samples and their control (p-value<0.1, Student’s t-test). [file Image_3.jpeg]

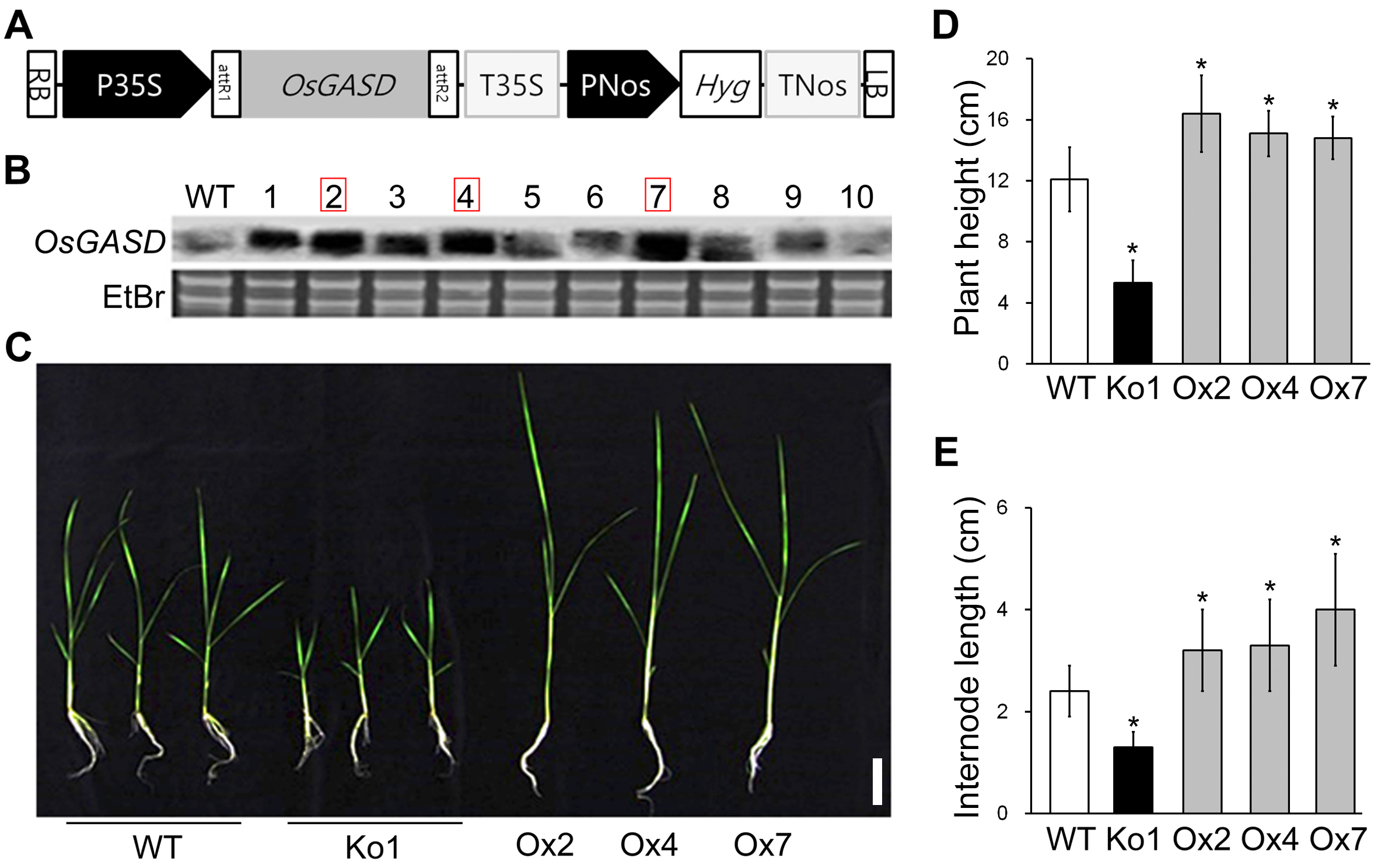

Supplement: Supplementary Figure 4 — Generation of transgenic rice plants overexpressing OsGASD gene. (A) Structure of the construct is show. LB, left border; RB, right border, HPT, hygromycin phosphotransferase. Northern blot analysis (B) of the OsGASD gene in the transgenic rice. Red boxes mark lines 2, 4 and 7 which were used in this study. (C-E) Analysis of overexpression of OsGASD plant (Ox) and osgasd knockout mutant (Ko1) phenotypes. (C) Morphology of 10-day-old wild-type (WT), overexpression of OsGASD plant (Ox) and osgasd knockout mutant (Ko1). Comparison of plant height (D) and internode length (E) were measured of the WT, Ox plants and Ko1 mutant. Error bars indicate SD. Asterisks indicate statistically significant differences between the corresponding samples and their control (p-value<0.1, Student’s t-test). Scale bar=1cm [file Image_4.jpeg]
